# Rationale, Implementation and Evaluation of Assistive Strategies for an Active Back-Support Exoskeleton

**DOI:** 10.3389/frobt.2018.00053

**Published:** 2018-05-25

**Authors:** Stefano Toxiri, Axel S. Koopman, Maria Lazzaroni, Jesús Ortiz, Valerie Power, Michiel P. de Looze, Leonard O'Sullivan, Darwin G. Caldwell

**Affiliations:** ^1^Department of Advanced Robotics, Istituto Italiano di Tecnologia, Genoa, Italy; ^2^Department of Informatics Bioengineering Robotics and Systems Engineering, University of Genoa, Genoa, Italy; ^3^Department of Human Movement Sciences, Faculty of Behavioural and Movement Sciences, Vrije Universiteit Amsterdam, Amsterdam Movement Sciences, Amsterdam, Netherlands; ^4^Department of Electronics, Information and Bioengineering, Politecnico di Milano, Milano, Italy; ^5^School of Design, University of Limerick, Limerick, Ireland; ^6^TNO, Leiden, Netherlands; ^7^Health Research Institute, University of Limerick, Limerick, Ireland

**Keywords:** exoskeleton, powered, manual material handling, strategy, myocontrol, electromyography

## Abstract

Active exoskeletons are potentially more effective and versatile than passive ones, but designing them poses a number of additional challenges. An important open challenge in the field is associated to the assistive strategy, by which the actuation forces are modulated to the user’s needs during the physical activity. This paper addresses this challenge on an active exoskeleton prototype aimed at reducing compressive low-back loads, associated to risk of musculoskeletal injury during manual material handling (i.e., repeatedly lifting objects). An analysis of the biomechanics of the physical task reveals two key factors that determine low-back loads. For each factor, a suitable control strategy for the exoskeleton is implemented. The first strategy is based on user posture and modulates the assistance to support the wearer’s own upper body. The second one adapts to the mass of the lifted object and is a practical implementation of electromyographic control. A third strategy is devised as a generalized combination of the first two. With these strategies, the proposed exoskeleton can quickly adjust to different task conditions (which makes it versatile compared to using multiple, task-specific, devices) as well as to individual preference (which promotes user acceptance). Additionally, the presented implementation is potentially applicable to more powerful exoskeletons, capable of generating larger forces. The different strategies are implemented on the exoskeleton and tested on 11 participants in an experiment reproducing the lifting task. The resulting data highlights that the strategies modulate the assistance as intended by design, i.e., they effectively adjust the commanded assistive torque during operation based on user posture and external mass. The experiment also provides evidence of significant reduction in muscular activity at the lumbar spine (around 30%) associated to using the exoskeleton. The reduction is well in line with previous literature and may be associated to lower risk of injury.

## 1. Introduction

Exoskeletons are wearable devices generally aimed at supporting physical tasks by generating appropriate forces on one or multiple human joints. There has been increasing interest in employing exoskeletons for workplace ergonomics to reduce the physical loads and risk of injury for workers carrying out demanding tasks. Work-related injuries not only increase the costs sustained by companies, but most importantly have a severe impact on the workers’ quality of life. Manual material handling, a common activity in various industrial sectors (e.g., car and aerospace manufacturing, logistics, construction), may be described as repeatedly lifting, moving and lowering objects for a relatively long time (e.g., some hours). During manual material handling large compressive forces over 5000*N* on the lumbar spine are generated, leading to a high risk of physical injury Norman et al. (1998); Coenen et al. (2014); OHSA (European Agency for Safety and Health at Work) (2000). Indeed, a large part of the cases of absence from work is associated to the spine, making the area most subject to disorders^1^. Guidelines for workplace safety and ergonomics often result in very strict limitations on the weights that can be handled depending on operating conditions such as frequency and posture Konz (1982); Waters et al. (1993); Garg (1995). These strict limitations give rise to opportunities for novel technical solutions, among which wearable exoskeletons have attracted great interest. A number of devices aimed at supporting the lower back have been designed as prototypes for research studies or developed as commercial products.

The design and assistive action of an exoskeleton strongly depend on its application. A basic distinction may be made between passive and active devices, based on whether the forces are generated by mechanical elements (e.g., springs) or by powered actuators (e.g., electromagnetic motors), respectively. The forces in an active exoskeleton are largely determined by how the forces from the actuators are controlled, which is done by the corresponding assistive strategy. A strategy consists of sensors that acquire meaningful information from the environment or the user as well as the program that turns the information into commands for the exoskeleton hardware. In order to generate appropriate assistive forces during the assisted task, the strategy needs to capture what the user needs or wants at a specific time. This idea is referred to as *following user intent*. By contrast, the behavior of passive exoskeletons is established at the design stage and cannot be adjusted during operation. Although sensors, computers and actuators certainly make the design of active exoskeletons more challenging compared to passive ones, it is generally considered that active devices hold the potential for superior versatility. The key to versatility and therefore a crucial component in their effectiveness is a suitable assistive control strategy. This aspect remains an open challenge due to the difficulty in acquiring meaningful information on user intent Lobo-Prat et al. (2014); Ansari et al. (2015); Toxiri et al. (2016); Young et al. (2017).

With particular reference to back-support exoskeletons, the challenge of appropriate modulation of forces in active devices is still relatively unexplored. Our overall research objective is to devise strategies that promote the physical effectiveness of the exoskeleton and integrate well with possible constraints and requirements of industrial applications. This manuscript starts by providing an overview on existing back-support exoskeletons from research and the market (Section 1.1), followed by a report of the relevant scientific literature on control strategies for exoskeletons with possible application to back-support devices (Section 1.2). Section 1.3 then summarizes the contributions of this study. The rationale for the proposed control strategies is given in Section 2, while Section 3 expands on their experimental evaluation. A final discussion is provided in Section 4.

### 1.1. Prior Work: Back-Support Exoskeletons

A review on existing exoskeletons and their reported effect on the physical work load was compiled in de Looze et al. (2016). In association with different back-support exoskeletons, reduction in physical work load has been quite consistently documented. Passive devices have led to reductions in muscular activity ranging between 10 and 40%, mostly in simplified laboratory scenarios [e.g., in Bosch et al. (2016); de Looze et al. (2016)]. These numbers establish convincing starting evidence of the potential effectiveness and encourage further development on back-support exoskeletons. In late 2017, at the time of writing the present manuscript, the review of de Looze et al. (2016) no longer provides a complete picture of the landscape of industrial exoskeletons. Over the last few years, a number of passive devices [including the commercial Laevo^2^ and BackX^3^ as well as research prototypes Babič et al. (2017)] have established a position within the community as more and more possible applications are found and tested. However, their actual daily use in the field has not been clearly demonstrated yet. On the other hand, new active exoskeletons have very recently made a strong appearance in the market (the Atoun Model A^4^ and the Hyundai H-WEX^5^), as companies have invested substantial resources in this sector. Due to their intrinsic versatility compared to passive systems, active exoskeletons hold the potential for even greater biomechanical benefits, although they are associated to significantly more complex designs. Their potential impact is still held back by substantial and open technological challenges, including the lack of effective control strategies capable of exploiting their versatility.

### 1.2. Prior Work: Control Strategies

While actuation technology is similar across many exoskeletons, a variety of control strategies can be found in literature. As the strategy largely determines the assistive action provided by an exoskeleton to its wearer, it typically needs to be designed for the specific target task. Possible ways to infer user intent and needs strongly depend on the target task. Every strategy has different advantages and drawbacks associated to the obtrusiveness of the sensors it uses and the active user participation it requires. In practical terms, the problem they address is to generate appropriate reference signals to control the speed, torque or impedance of the actuated joints over time Tucker et al. (2015). This aspect remains an open challenge due to the difficulty in acquiring meaningful information on user intent Lobo-Prat et al. (2014); Ansari et al. (2015); Toxiri et al. (2016). A number of reviews on control strategies have been published in recent years Tucker et al. (2015); Yan et al. (2015); Chen et al. (2016); Ahmed et al. (2016); Young et al. (2017), but little material is available relevant to active back-support exoskeletons. Although the following examples are applications on lower- or upper-limb exoskeletons, it is still helpful to discuss them with focus on their advantages, disadvantages and potential applicability to back-support exoskeletons. For example, it has become common to distinguish between *direct* and *indirect* strategies, depending on whether information is acquired from the user (e.g., biosignals) or from the environment (e.g., joints motion or ground reaction force), respectively.

#### 1.2.1. Indirect Strategies

Commanding an exoskeleton based on the motion of relevant body segments is particularly suited for cyclic tasks such as walking. In this case, an exoskeleton would attempt to match the cadence and reproduce a set of predefined assistive actions in loops. Relevant examples are presented in Ronsse et al. (2011); Giovacchini et al. (2015); Ruiz Garate et al. (2017) with applications in elbow and hip assistance. It is helpful to highlight here that sensors for joints orientation are usually well integrated in the exoskeleton and are therefore little obtrusive. However, reproducing profiles in loops is less suited for non-cyclic tasks. Alternatively, measurements of interaction forces have been used as inputs for assistive strategies. The ground reaction force (GRF) is used in combination with knee joint angle on the RoboKnee to provide assistance against gravity Pratt et al. (2004). A similar approach is taken on a different and somewhat unique device, the Honda Walking Assist Device Ikeuchi et al. (2009). With focus on lifting objects rather than walking, GRF is used to generate commands for a wearable knee exoskeleton Saccares et al. (2017) and for a ground-based robotic arm Kim et al. (2018). One of the issues typically associated to these measurements is the obtrusiveness of the sensors that measure the GRF. In some cases they may limit movement, in others they may require being worn inside the user’s or special shoes. In industrial applications, where operators may be demotivated by the need to wear extra equipment, these limitations may compromise user acceptance. BLEEX is a well-known lower-limb exoskeleton for performance augmentation in walking long distances with heavy loads Kazerooni et al. (2005). The idea is that the load is part of a backpack and its weight redirected to the ground via the exoskeleton structures. The actuated joints, strapped onto the user’s leg segments, are commanded to follow the user movements with the lowest interaction forces possible. This is indeed the key feature of this strategy, which makes it unsuitable for devices designed to apply substantial assistive forces onto the user to reduce loads on specific parts, as is the case in the this study as well as with the HAL Lumbar Support Hara and Sankai (2010). One of its control modes is based on a well-integrated measure of posture. Assistance is then provided as a force proportional to the inclination of the torso. This is of particular interest in the present context.

#### 1.2.2. Direct Strategies

Surface electromyography (EMG) is perhaps the most representative technique for direct control of exoskeletons^6^. This technique is based on measuring very small electrical signals that are directly associated to muscular activity. Applications for monitoring purposes are not of interest here [see Wehner (2012); Hakonen et al. (2015) for a complete overview], thus it is considered most helpful to first outline its uses for controlling exoskeletons, while practical limitations are discussed below.

A major trend in the literature is to employ models to map the measured muscular activity into exerted muscle force and command an exoskeleton accordingly, e.g., regulating the speed or force at its joints. Some examples can be found in Rosen et al. (2001); Hayashi et al. (2005); Fleischer et al. (2008). Using similar models, Karavas and colleagues estimated human joint stiffness by reading the activity of two antagonistic muscule groups at the knee Karavas et al. (2013). In that study, the mechanical stiffness displayed by a knee exoskeleton was controlled correspondingly. An important limitation of model-based approaches is that they require frequent, subject-specific identification of model parameters, which may not be practical in field scenarios outside laboratory environment.

In contrast with previous literature supporting the need for accurate models, recent studies have successfully implemented more straightforward approaches whereby EMG amplitude is more directly (e.g., proportionally) mapped into a reference force/torque for an exoskeleton joint. A study on the HAL Lumbar Support tested this *proportional myoelectric control*Hara and Sankai (2010) (which will be discussed further in this paper). Lenzi et al later highlighted its relevance, proposing that an approximate measure of muscular activity may indeed be sufficient to control assistive exoskeletons Lenzi et al. (2012). An additional study by the same group successfully showed that a device assisting one joint may even be controlled via a muscle acting on a different joint, as long as the two muscle groups are activated in coordination during the target task Grazi et al. (2015). These findings encourage further research towards the simplification of direct control strategies for wearable robots, thus making their adoption more likely and impactful on the wide public. Recent applications of proportional myoelectric control are also described in Young et al. (2017); Meattini et al. (2017).

The traditional complexity of EMG setups makes this technique typically unsuitable outside research laboratories and in industrial applications for which quicker and simpler solutions would be more appropriate. In this respect, the solution proposed in this study and described in the next section represents a substantial improvement.

### 1.3. Contributions of This Study

This paper addresses the challenge of devising suitable control strategies for the modulation of the assistive forces on an active back-support exoskeleton. The contribution with respect to the available literature articulates into (a) presenting the requirements for control strategies based on the task dynamics, and (b) the implementation of an EMG-based control strategy that fits applications in industrial scenarios more practically than traditional setups.

The approach starts from the description of the target physical task and of its dynamics. The analysis highlights the main parameters that determine the need for assistance and should thus be taken into account by appropriate control strategies. The devised strategies are evaluated in terms of the assistive behavior that they generate during experimental trials. Additionally, data on the resulting physical effectiveness in terms of reduced muscular activity is provided.

## 2. Methods

This section presents the proposed assistive strategies, including an overview of the platform for which they were developed as well as the rationale that guided and motivated their development.

### 2.1. Platform: Exoskeleton Prototype

The device used in this work is an active back-support exoskeleton. Its development was supported by the EU-funded FP7 project Robo-Mate^7^ Stadler et al. (2017) and has later continued via national funding by INAIL (the Italian Workers’ Compensation Authority). This section describes the details of a revised second version, named Mk2b.

#### 2.1.1. Structures

**Figure 1:**
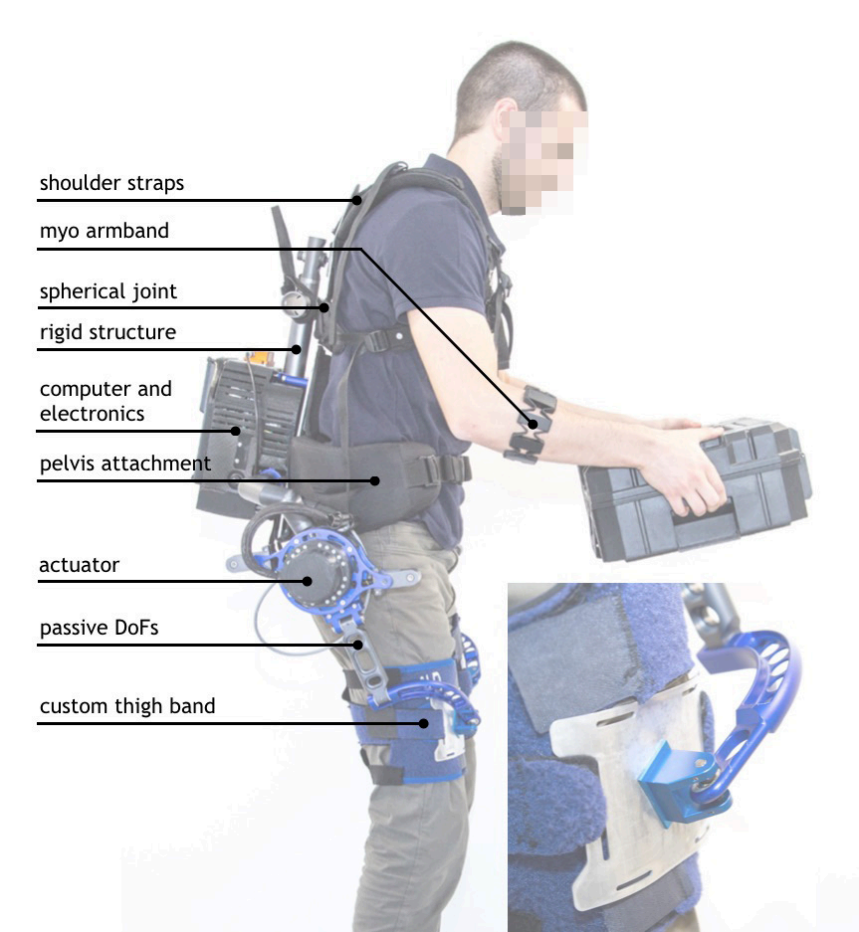
Side view of the *Mk2* prototype. This image is being published with written informed consent of the depicted individual.

The prototype, represented in Figure 1, spans the torso and upper legs similarly to most of the devices described in Section 1.1. On the torso, it is attached via parts of a commercial backpack, including shoulder straps with front clip, a wide waist band, and a padded rigid plate at the lower back. Custom Velcro-bands to fix the leg links to the thighs were sewn in-house. Attached on the rigid back plate is a custom-designed rigid frame that holds the two actuators in place, one on each side lateral to the hip joint and approximately aligned with its axis of flexion-extension. During the donning procedure an assistant attempts to align the actuator at the hip, and subsequently the multiple adjustment straps are used to distribute weight and pressure to the user’s preference. Each actuator generates torque between the rigid frame and the corresponding thigh link. The torque is approximately limited to the sagittal plane. The leg links connecting each actuator to the corresponding thigh band are endowed with a set of five passive degrees of freedom Toxiri et al. (2015). Additionally, the shoulder straps are connected to the rigid frame via a spherical joint providing three additional passive degrees of freedom. These ensure that user movements are unhindered (e.g., twisting the torso; hip abduction and adduction; hip internal and external rotation) and as a result promote comfort.

#### 2.1.2. Actuators and Electronics

Each actuation unit includes a brushless DC motor coupled to a compact reduction gear. The joint torque produced by the actuator is measured via a commercial, strain gauge-based joint torque sensor placed between the gear output and the link connecting with the user’s upper leg.

#### 2.1.3. Two-Level Control Scheme

**Figure 2:**
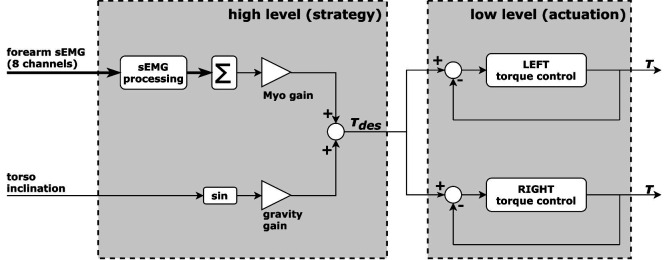
The implemented control scheme, articulated in two levels. The low level regulates the torque output at the actuators. The high level corresponds to the assistive strategy and is responsible for the extent and timing of the generated assistance.

The control scheme is structured on two levels, as depicted in Figure 2. The general goal of this scheme is that the user is free to move as intended and additionally experiences substantial assistive forces with appropriate timing and extent. This concept has been referred to as *following user intention*. On the low level, a closed-loop torque controller is in charge of tracking the reference torque signal at each actuator^8^. A high-level strategy establishes the necessary amount of assistive torque and generates a reference signal accordingly. Note that the same torque reference signal is sent to each actuator, resulting in twice that torque to be provided to the user as physical assistance.

### 2.2. Rationale for Strategies: Biomechanics of Lifting

**Figure 3:**
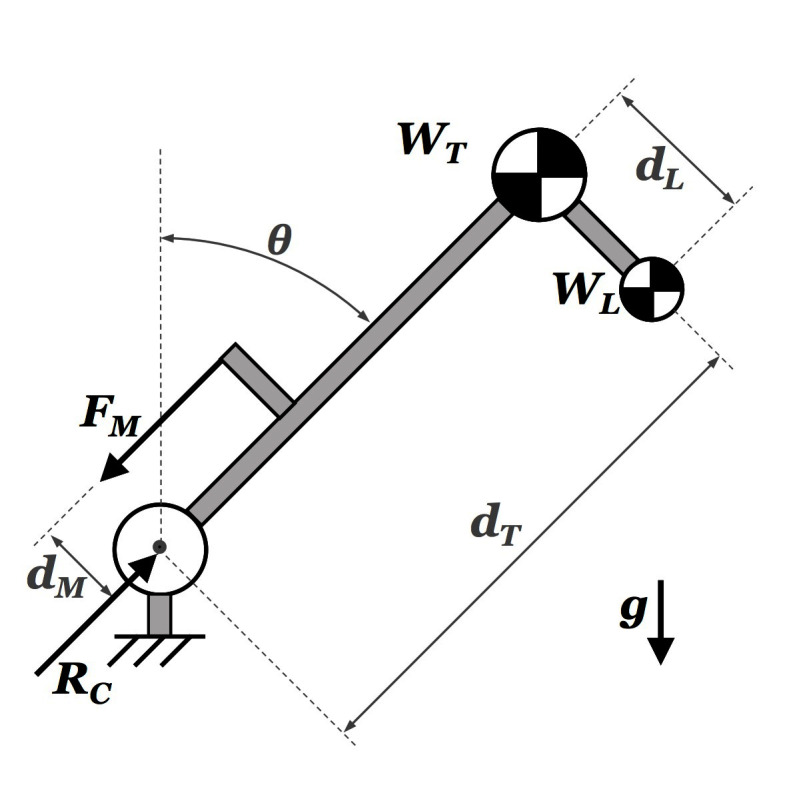
Simplified model of the compressive loads on the lumbar spine. The torso, represented with mass *W_T_*, is articulated to the pelvis via a rotational joint representing the low back. The spinal muscles generate force *F_M_* at a *d_M_* distance from the joint, contributing to compression forces *R_C_*. The external object is represented with mass *W_L_*.

**Figure 4:**
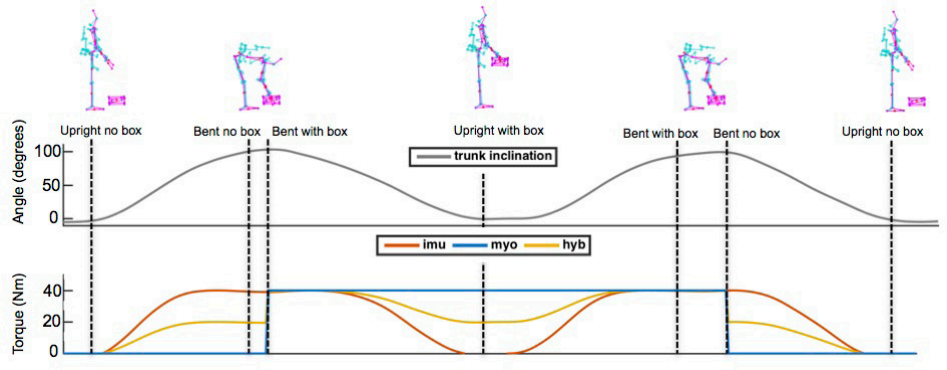
This simplified illustration further describes the idea behind the implemented control strategies. The top plot displays the inclinationg angle of the torso over time, during the execution of the simplified task illustrated by the stick figures above. The bottom plot displays the corresponding torque reference signals generated by the three different assistive stragies. In red, *imu* follows the inclination of the torso regardless of whether the user is holding the object. The *myo* mode (blue line) is represented as only switching on when the user holds the object. In yellow, *hyb* displays a combination of the two behaviors, in which each branch contributes to half of the generated reference torque.

**Figure 5:**
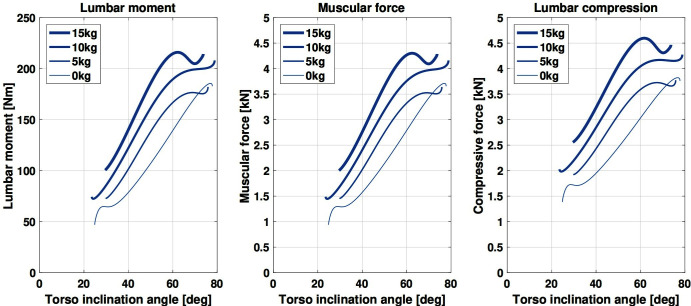
Lumbar moment (left) computed via inverse dynamics, applying real motion data to the model in Figure 3. As a consequence, the muscular force *F_M_* (center) and joint reaction force *R_C_* (right) are calculated. The three show similar trends, depending on two key factors: **(A)** the *orientation of the upper body*, and **(B)** the *mass of the object* being handled.

A simplified two-dimensional model is employed to gain quantitative understanding of the biomechanics of the lumbar spine during the target task. The model, illustrated in Figure 3 and detailed in Toxiri et al. (2015), represents the lumbar spine as a rotational joint connecting the torso mass *W_T_* to the pelvis, which is simplified as attached to ground. The spinal muscles, responsible for back extension, are represented as generating a force *F_M_* parallel to and posterior to the spine (at a *d_M_* distance). The reaction force *R_C_* at the joint captures the lumbar compressive loads, which the exoskeleton aims to reduce. The external object is represented by an additional (variable) mass *W_L_*, rigidly connected to the upper body. Human motion data^9^ (from the physical task illustrated in Figure 4) applied to this model allows the estimation of the net lumbar moment via inverse dynamics (Figure 5, on the left). This estimate is then used to compute the corresponding muscular force (Figure 5, center) based on an approximated, fixed lever arm. Figure 5, on the right, shows the estimate of the resulting compressive force acting on the lumbar spine while handling objects from 0 *kg* to 15 *kg*.

Two key factors appear to affect lumbar moment, muscular force and lumbar compression in the same way: (a) the *orientation of the upper body*, and (b) the *mass of the object* being handled. The compression increases with the orientation angle, reflecting a corresponding increase in muscular activity. Indeed, greater forces at the *erector spinae* muscle group are necessary to balance the moment generated by gravity acting on the user’s upper body and external mass. As a consequence, greater compression is associated to increasing object mass. Similarly to above, the spinal muscles activate to balance the increasing load and in turn larger compressive reaction forces are generated on the lumbar joint.

In order to promote appropriately timed and modulated physical assistance based on the considerations, the two factors were taken into account for the design of the assistive strategy for the exoskeleton.

### 2.3. Proposed Assistive Strategies

This section described the implementation of different strategies implemented on the exoskeleton. The first and second strategies (Sections 2.3.1 and 2.3.2) each reflect one of the two factors described in the previous section. The third one (Section 2.3.3) represents a more general case and is implemented as a combination of the first two. Figure 4 provides an illustration of their working principle. The gray line in the top plot shows how the torso inclination angle varies during the task (see also the stick figures at the top). The bottom plot shows an approximation of the torque reference signals generated by the different strategies, represented in different colors. The following sections provide a detailed description of their implementation.

#### 2.3.1 Inclination-based

This strategy implements an approximate version of what on a robotic arm would be known as *gravity compensation*. The idea is to relieve the user from the effort spent on holding the torso link of the exoskeleton as well as his/her own torso. The implementation does not attempt to precisely estimate the mass properties of the user’s upper body and exoskeleton links to exactly compensate the effects of gravity. The gain corresponding to this branch may be fixed or adjusted to suit individual preference and/or comfort.

(1) $$\tau_{des,imu}=K_{imu}\cdot sin\left(\theta_{trunk}\right)$$

The torso inclination angle is acquired via an xSens MTi-30 AHRS inertial measurement unit (IMU) attached at the back the exoskeleton rigid structure.

#### 2.3.2. Proportional Myocontrol

The second strategy is based on surface electromyography and targets the second of the two factors described in Section 2.2. Typically, the activity of one or more muscles acting on the assisted joint would be acquired, so that the same physical activity can be accomplished with less muscular activity. Examples from recent literature are Young and Ferris (2017), in which the activity of gluteus and quadriceps was used to modulate assistance at the hip, and Meattini et al. (2017), in which elbow flexion is assisted with forces modulated on biceps activity. The closest example to this strategy is reported in Hara and Sankai (2010). In that study, HAL Lumbar Support assisted hip and back extension proportionally to the activity of the spinal muscles. By contrast, the controller presented here generates reference values for the assistive torques proportionally to the activation of the forearm muscles. As anticipated, the concept of assisting a muscle based on the activity of a different one was first explicitly described in Grazi et al. (2015). This option is suitable if during a given task the two muscle groups activate in coordination, and if measuring the activity of the main muscle is technically challenging in practice while the secondary muscle is more easily accessed.

The activity of the forearm muscles is recorded by the electrodes on the Myo armband (a description of this device is provided below). As opposed to the activity of any specific muscle at the forearm, their overall activity is considered. This represents a big practical advantage as it eliminates the need for careful electrode placement. Thus, the sum of the eight rectified signals acquired was considered as an indication of grip strength, and therefore connected to the mass of the object being held. The signal generated at this stage is loosely referred to as *myo*. It is normalized by a maximum value, which is acquired during a preliminary calibration phase or readjusted at any other time if necessary. The maximum value may for example capture the activation corresponding to the heaviest object one expects during a task (as is done in the experiments described in Section 3).

The control scheme generates the corresponding component of the assistive torque as proportional to the normalized *myo* signal, via a gain that determines to what extent the exoskeleton contributes to the task, and thus potentially reduces the user’s effort to accomplish it.

(2) $$\begin{array}{c} \tau_{des,myo}=K_{myo}\cdot EMG_{sum,norm}\\ EMG_{sum}=\sum EMG_{i}\\ EMG_{sum,norm}=EMG_{sum}/max\left(EMG_{sum}\right) \end{array}$$

#### 2.3.3. Hybrid

The more general case of the strategy illustrated in Figure 2 is referred to as *hybrid* strategy. In this general case, the two inclination-based and EMG-based branches are active at the same time, each of the two regulated by the corresponding control gain, as follows:

(3) $$\tau_{des,hyb}=K_{imu}\cdot sin\left(\theta_{trunk}\right)+K_{myo}\cdot EMG_{sum,norm}$$

In principle, it is possible to adjust *K_imu_* and *K_myo_* for each user and/or tasks to best meet personal preferences and task conditions.

#### The Myo Armband

**Figure 6:**
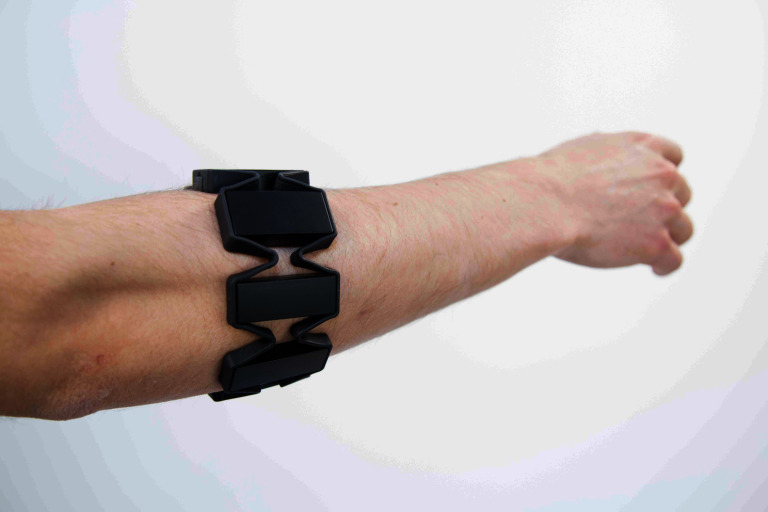
The Myo armband is a commercial device that integrates eight pairs of electrodes for dry sEMG acquisition. The device is powered by built-in batteries and is capable of sending the signals over a Bluetooth connection.

As part of our approach, muscular activity at the forearm is measured via an inexpensive commercial device based on surface electromyography. The Myo gesture control armband^10^ (shown in Figure 6) offers eight pairs of dry electrodes, equally spaced around the band, typically worn on the forearm. This device is convenient for a number of reasons, besides its affordability. The surface electromyography on the Myo uses dry electrodes. This solution requires no skin preparation nor pre-gelled disposable electrodes. Considering the target task of lifting object, wearing a compact armband is less invasive than the corresponding setup on the low back underneath the clothes. In fact, this would require an additional person for the skin preparation and electrode positioning, besides potentially limiting the user’s movement and resulting in poor signal quality due to mechanical interference with the exoskeleton structure and/or straps. As an additional benefit, the device sends out data via a practical wireless communication and is powered by built-in batteries. The sEMG signals acquired on the forearm were preprocessed on the Myo armband itself. A custom script on the main on-board computer collected the eight filtered and rectified signals and made them available to the main program controlling the exoskeleton. For the purpose of control, the signals were summed and further low-pass filtered with a cut-off frequency set to 3 Hz, chosen empirically as a trade-off between physical comfort and responsiveness.

## 3. Evaluation

In this study, the evaluation focuses on different aspects. Firstly, it is important to verify that the implementation of the proposed strategies follows the intended design, as illustrated in Figure 4. This is expanded in Section 3.2. On the other hand is the physical effectiveness, i.e., the effect on the user’s body in terms of biomechanics, must be as intended. Relevant experimental data is presented in Section 3.3. For an exoskeleton to be successfully adopted in the field as a product, there are additional aspects that go beyond the scope of this study. For instance, the device must be accepted well by users, who feel encouraged to use it, and must be affordable and integrate well with the existing infrastructure, so that employers are motivated to purchase it.

When studying the physical effectiveness of exoskeletons for injury prevention, a common focus emerging from the review in de Looze et al. (2016) is on the reduction in muscular activity. This is often a case of convenience, as muscular activity can be quite readily measured in a research laboratory with non-invasive technologies (although not free of complications). On the other hand, joint loading can only be estimated indirectly^11^ and requires the use of additional technology (e.g., motion capture setups) and musculoskeletal models, which results in substantially more time-consuming testing procedures. In relation to the lumbar spine, muscular activity is considered for its close relationship with joint loading, as described in Section 2.2. Therefore, a significant reduction in muscular activity at the spine is reasonably associated to an reduction in the corresponding compressive loads. Experiments to assess the physical effectiveness of exoskeletons are often performed in controlled laboratory settings. For back-support exoskeletons, the tasks typically involve some type of static and dynamic lifting meant to represent the activities carried out in the workplace. As the objective is to capture the effect of the exoskeleton, the different experimental conditions capture whether the task is performed with or without the assistance of the device. Another important variable that applies to active exoskeletons is the strategy by which they are controlled, when more than one is available (as is the case in this study). Additional conditions may consider different loads and lifting techniques (i.e., squat or stoop).

In this paper, the motivation and description of different assistive strategies is supported by experimental data reporting their effect on the users in terms of muscular activity. The experimental campaign was carried out at the Vrije Universiteit Amsterdam in October 2017 using the Mk2b prototype. The experiment attempted to replicate the scenario of the target task, with the goal of observing differences between the different strategies described above.

### 3.1. Experimental Protocol

Eleven healthy young males (age 25.0 years, SD 6.9 years, weight 70.9 kg, SD 8.8 kg, height 1.77 m, SD 0.06 m) participated in the experiment. None of the participants had a history of low-back pain. The experiment was approved by the local ethics committee. After signing an informed consent, each subject was instructed to complete a lifting and lowering task in different conditions, described as follows:

*no exo*: no exoskeleton is worn; 

*imu*: the exoskeleton assists based on the inclination strategy (Section 2.3.1), with *K_imu_* = 20; 

*myo*: the exoskeleton assists based on the sEMG strategy (Section 2.3.2), with *K_myo_* = 20; 

*hyb*: the exoskeleton assists based on the hybrid strategy (Section 2.3.3), with *K_imu_* = *K_myo_* = 10.

The *no exo* condition was performed first in all cases^12^, while the order of the remaining three was randomized. As part of the task, each condition started in an upright position (Figure 4 provides a helpful illustration). The participant would then bend over, reach and grasp an object from mid-shin height and take it up to an upright position. The participant would then bend over once more, place the object back into its original position, go back to an upright posture, and repeat this procedure for a total of three repetitions. This segment was executed twice, starting with a 7.5 kg object and then with a 15 kg object, so that for each condition the participant would lift and lower a total of six times. No minor or major injury occurred during the experimental campaign. No instructions on a specific lifting technique (i.e., stoop or squat) or speed was given to the participants. To minimize the impact of fatigue, subjects were allowed to take short breaks between the different lifting conditions. The object consisted of a container with handles, loaded with known weights that could be removed to accomodate for the different loads during the experiment. For the purpose of calibrating the corresponding control strategy, the *myo* (forearm) sEMG signal was normalized for each participant during a preliminary calibration session, during which a 15 kg object was held for one second against gravity.

### 3.2. Reference Torque Profiles

**Figure 7:**
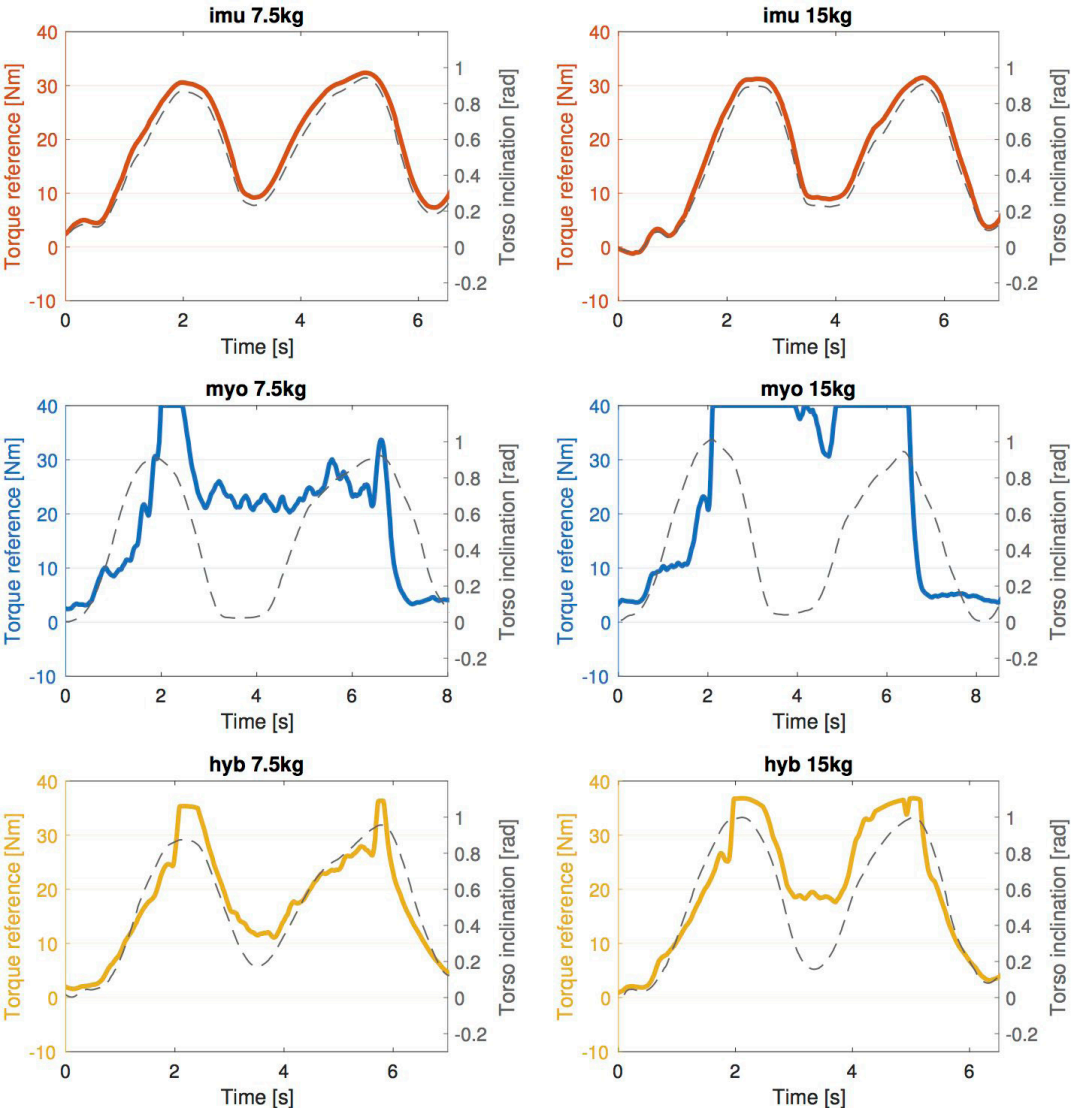
Torque reference profiles generated by each of the three strategies. Data is shown for one subject, for illustration purposes. The red signal mostly overlaps with the torso orientation, while the blue lines in the second row is high between the peaks, and the value is larger for heavier load (on the right). In the third row, the *hyb* reference displays an intermediate behavior between the two above.

As anticipated, this part expands on whether the torque reference profiles generated by the different strategies correspond to the intended design, illustrated in Figure 4. Experimental data from one subject is shown in Figure 7. The total reference torque is plotted together with the corresponding signal for torso inclination (dashed grey lines), to relate with the movement of the user. It is interesting to observe how the torque reference generated by each of the three strategies differs in terms of timing and extent of the assistance provided to the user. In the top row, the red profile associated to the *imu* strategy produces a reference that mostly overlaps with the torso orientation both on the left and the right plot, corresponding to the 7.5 and 15 *kg* object respectively. In the second row, the blue signal represents the reference generated by the *myo* strategy. The reference torque increases corresponding to when the user picks up the box (left peak in torso inclination angle), and decreases again when the box is released (right peak in torso inclination angle). The bottom row shows intermediate trends between the two above. The yellow reference torques follow the orientation closely, although their values in between peak pairs are larger for the heavier object.

### 3.3. Muscular Activity

Standard laboratory sEMG equipment was fitted to measure the activity of left and right spinal muscles (*iliocostalis*) following SENIAM guidelines.

#### 3.3.1. Data Analysis

The sEMG signals were rectified and filtered according to standard practice (low-pass frequency at 2.5 *Hz*), and ultimately normalized to the maximal voluntary contraction (M.V.C.) acquired during a preliminary procedure. For each condition and object mass the peak activity was considered, ultimately using the average value between left and right side. Two one-way repeated measures ANOVAs were performed for each of the weights (7.5 and 15 kg) with support condition as within-subject factor (no-exo, imu, myo, hyb). Bonferroni corrected post-hoc tests were performed after a significant main effect of support condition was found. A significance level of *p* < 0.05 was used. 

#### 3.3.2 Results

**Figure 8:**
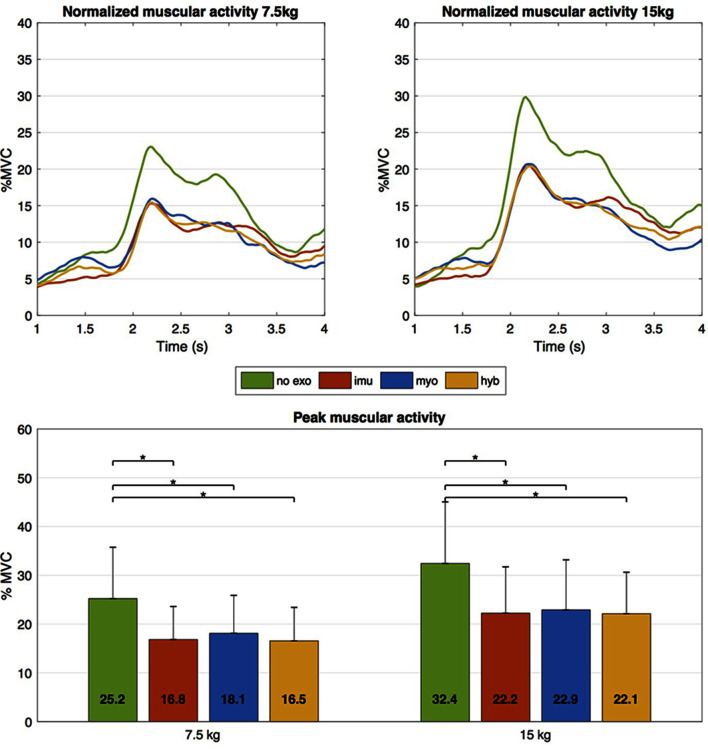
Muscular activity for the different conditions. At the top, averaged EMG profiles across all subjects are shown. In all cases, wearing the exoskeleton is associated to decreased muscular activity, although none of the three strategies leads to overall larger reduction than the others. In terms of peak activity, the data is summarized at the bottom, where peaks (average and SD across all subjects) are shown for the different conditions. Significant percentage reductions (*p* < 0.05) for the three strategies range between 28 and 35%.

The results for muscular activity are shown in Figure 8. At the top, the activity profiles are shown as averaged across all subjects, together with the corresponding profiles of torso orientation (dashed lines). With respect to the no exo (green) condition, reduced activation of the spinal muscles is observed in all cases. More in detail, the average profile associated to the imu (red) control leads to the lowest activation during the first phase (before 2.0s), before the user reaches the object. The same holds for the final phase (around 6.0s), when the person is standing back up after releasing the object. This consideration is valid for both loads: 7.5 kg (left plot) and 15 kg (right plot). By contrast, myo (blue) is associated to lowest muscular activity in the phase (around 3.5s). This time corresponds to the second descent phase when the user is holding the object and, from an upright position, bends forward again to take the box back down. At the same key times, the yellow profile representing the hyb condition displays intermediate values with respect to the two above. For both weights a significant main effect of support condition was found. The bottom part of Figure 8 shows the activity peaks averaged across all subjects for the different conditions. Similarly to average activation profiles, peak activation is also reduced by all three strategies, for both loads. With respect to the no exo condition, significant percentage reductions (*p* < 0.05) in the peaks ranging from 28% to 35% were observed.

## 4. Discussion

The biomechanical analysis in Section 2.2 highlighted the two main factors that determine low-back loading, and therefore the need for physical assistance, during manual lifting tasks. In order to generate appropriate assistance with the active back-support exoskeleton described in Section 2.1, the *imu* and *myo* strategies were designed to address the two factors separately. Each strategy is associated to advantages and drawbacks that need to be considered in the context of practical use. The *imu* strategy has the advantage of only relying on very well-integrated hardware, entirely unobtrusive to the user. The disadvantages are connected to its inability to modulate the assistance to varying external loads. Therefore, it may be a good solution by itself when the load is known in advance, or for the specific case of supporting static postures. By contrast, the capability of the sEMG-based strategy to maintain substantial assistance whenever the user holds an object (and proportionally to its mass) is considered a beneficial feature for an exoskeleton designed for repeated lifting. While data from the specific subject shown in Figure 7 executed the task slightly slower with the *myo* compared to the other strategies, the authors do not consider this difference to be of interest with respect to the present discussion. Compared to the state of the art in sEMG-based control, the implemented strategy has the strong advantage in terms of practical applicability. This is due both to the very unobtrusive and inexpensive hardware it makes use of, as well as to the minimal need for calibration. For instance, a similar approach described in Hara and Sankai (2010) was based on a sEMG measurements on the muscles at the lower back, which is considered relatively invasive for an industrial application as well as prone to artifact due to the contact with the exoskeleton structures. By contrast, the forearm device used in our implementation is easily and autonomously worn and set up. The authors expect the state of the art on strategies for active back-support exoskeletons to advance substantially in the near future, as more research and development focuses on industrial applications.

An important difference between the two strategies is connected to the possibility of scaling the assistive forces up. As mentioned, the *imu* strategy is suited for known loads. The weight of the load may for example be used to scale the assistance up or down by adjusting the corresponding gain *K_imu_*. However, larger gain would lead to increasing forces also when no object is being held and thus no (or only low) assistance is necessary, generating unwanted hindrance. Conversely, larger *myo* gains *K_myo_* would lead to greater forces corresponding to heavier loads, therefore according to an increased need for assistance. This aspect makes this strategy of particular interest considering the possible future development of actuators, capable of generating larger forces at the required speeds. Based on the above, the *hybrid* strategy may be the best overall solutions as it potentially combines the advantages of the two strategies above. Adaptation to varying lifting speed, which affect back loading due to dynamic effects, may be provided in future implementations by using signals already available in the current platform. Practically, the above means that the same exoskeleton may readily adapt to different tasks wherein one or the other branch may be more or less necessary. For example, in a factory, the exoskeleton may be used to assist multiple, potentially different, tasks by selecting an appropriate strategy from an available set, and/or further adjusting the parameters of each based on specific needs.

The results on muscular activity indicate that the use of the exoskeleton, controlled by any of the proposed strategies, leads to reduced activation of the spinal muscles. This is positively associated to reduced compression forces at the lumbar spine and therefore suggests potentially lower risk of musculoskeletal injuries during repeated lifting activities. The numbers found in this study (percentage reductions between 28 and 35%) are in line with those indicated in the existing literature [see de Looze et al. (2016)], which confirms the effectiveness of the specific prototype and encourages further research work aimed at more accurate understanding of the physical effects. With respect to the strategies, at this point none of the three appeared to prevail over the others in terms of greatest reduction in peak muscular activation. Indeed, in this implementation they generate approximately the same assistance at the moment of peak loading. This aspect needs to be looked into in more detail to guide future design stages.

Besides the device’s physical effectiveness, individual preferences should also be considered to promote the use of exoskeletons. In this direction, it may be valuable to provide each user the ability to adjust, within certain safety limits, the control parameters (*K_imu_* and *K_myo_*) to promote one’s own comfort. The proposed device would easily implement this possibility, as it is controlled via on-board computers.

### 4.1. Limitations of This Study

The simplifications in the physical task carried out by the participants (see Section 3.1) do not currently allow to draw conclusions on a number of aspects. For example, the effect of muscular fatigue on muscle activation while using the exoskeleton cannot be observed in such short trials and would require longer experiments. Also, this study was not designed to observe the effect of the exoskeleton on joints other than the lower back that may be affected, such as the knee. Evidence excluding extra loading on the knee using this device was presented in Huysamen et al. (2018).

Additionally, the device used in this study is still a research prototype. Although it has also been used in preliminary pilot trials in industrial settings (outside the scope of the present study), it should be taken as a non-final prototype, whose effect may improve following further work on its implementation. For example, in the current version the electrical power for the actuators is delivered by an external supply via a cable, which limits the usability of the device to confined, uncluttered spaces where electrical power is available. In this respect, battery power for improved mobility and autonomy is part of the plans for future technical development.

## 5. Conclusion

Active exoskeletons are potentially more effective and more versatile than passive ones in assisting physical tasks. Their potential is dependent on appropriate assistive strategies, which modulate the assistance provided during the task to maximize effectiveness (known as *following user intent*). This paper addressed the open challenge of designing appropriate strategies for an exoskeleton reducing spinal loads during manual handling. By studying the biomechanics of the physical task, two key factors were identified (related to user posture and external mass, respectively), and a corresponding strategy devised and implemented. One of them, based on surface EMG, may represent a significant step forward as it enables the practical use of a meaningful but otherwise challenging signal to use outside a laboratory setting. This strategy is also more suitable to devices featuring stronger actuators, which may be available in the near future and could lead further reducing the musculoskeletal loading.

An experimental campaign aimed at evaluating the implementation of the strategies was performed involved 11 participants carrying out a simulated lifting and lowering task in different conditions. The resulting data validates the implementation of the strategies, which generate assistive behaviors as originally devised. This aspect indirectly supports the superior versatility of an active exoskeleton. Such device, with its ability to implement and modulate different strategies, may in fact target multiple tasks, in comparison with a set of task-specific passive devices. Additionally, statistically significant reduction in the activity of the relevant muscles provides evidence of the physical effectiveness of the prototype presented in terms of decreased lumbar loads and therefore risk of injury.

## Ethics Statement

This study was carried out in accordance with the recommendations of The Scientific and Ethical Review Board (VCWE) of the Faculty of Behavior & Movement Sciences, VU University Amsterdam with written informed consent from all subjects. All subjects gave written informed consent in accordance with the Declaration of Helsinki. The protocol was approved by the VCWE on September, 13th 2017 with reference number VCWE-2017-138.

## AUTHOR CONTRIBUTIONS

ST helped devise and implement the strategies, devise and run the experiment, and draft the manuscript. AK helped devise and led the experiment, and helped draft the manuscript. ML helped devise and run the experiment. JO helped devise the experiment and draft the manuscript. VP helped devise and run the experiment. MPL, LOS and DC helped devise the experiment and obtaining funding and resources. 

## Conflict of Interest Statement

The authors declare that the research was conducted in the absence of any commercial or financial relationships that could be construed as a potential conflict of interest.

## Footnotes

1 In 2016, the Italian Workers’ Compensation Authority (INAIL) reports over 31000 new cases of absence from work due to musculoskeletal disorders (about 68% of the total cases of absence). A large part of these (42%) are associated to the spine, making it the area most subject to disorders. Among the causes, spine disorders are also associated to longer absences compared to the overall average. Data are taken from INAIL’s open database, available at http://dati.inail.it/opendata/ (in Italian).

2 http://www.laevo.nl/

3 http://www.suitx.com/backx

4 http://atoun.co.jp/products/atoun-model-a

5 https://www.hyundai.news/eu/technology/hyundai-motor-leads-personal-mobility-revolution-with-advanced-robots/

6 Electroencefalography (EEG), aimed at measuring brain electrical activity as reflected on the surface of the scalp, is a comparatively more invasive technique, at this stage still more suited for use in the laboratory. It is thus not considered in this work.

7 http://www.robo-mate.eu

8 A description of the low-level controls is outside the scope of this article and therefore omitted.

9 Thanks to the Netherlands Organisation for Applied Scientific Research (TNO) for providing the data.

10 http://www.myo.com

11 Instrumented vertebral implants for in-vivo measurement exist, but are rather invasive and therefore not considered here.

12 This simplification to the protocol was made to minimize the risk of unintended changes in the experimental setup, particularly associated with unwanted displacement of the sEMG electrodes when donning and doffing the exoskeleton.
